# A randomised controlled trial comparing internet-delivered cognitive behavioural therapy (iCBT) with and without main carer access versus treatment-as-usual for depression and anxiety among breast cancer survivors: Study protocol

**DOI:** 10.1016/j.invent.2021.100367

**Published:** 2021-01-21

**Authors:** Selin Akkol-Solakoglu, David Hevey, Derek Richards

**Affiliations:** a Aras an Phiarsaigh, School of Psychology, Trinity College Dublin, Dublin, Ireland; b Clinical Research and Innovation, SilverCloud Health, Dublin, Ireland

**Keywords:** Cognitive behavioural therapy, Internet treatment, Depression, Anxiety, Breast cancer survivor, Carer access

## Abstract

**Background:**

Depression and anxiety are common problems among breast cancer survivors. Carer support is one of the most important determinants of women's psychological wellbeing. Survivors' distress can be alleviated by giving carers access to survivors' evidence-based treatment, which will help carers understand what survivors have been going through and help survivors feel more supported. Given the limited access to evidence-based treatments, an adapted internet-delivered cognitive behavioural therapy (iCBT) intervention for breast cancer survivors, but also open for carers' access, has the potential to decrease survivors' depression and anxiety symptoms and improve cancer-related communication and relationship quality between survivors and carers.

**Objectives:**

This study evaluates (1) the effectiveness of a guided iCBT intervention for depression and/or anxiety symptoms among breast cancer survivors with and without main carer access, and (2) the acceptability and satisfaction with the iCBT programme.

**Method:**

In this pilot study comparing the effectiveness of an adapted 7-week iCBT without main carer access against the iCBT with main carer access and treatment-as-usual control, 108 breast cancer survivors will be recruited and then randomised to either (1) treatment (*n* = 72) or (2) treatment-as-usual control group (*n* = 36) with a 2:1 ratio. The participants in the treatment group will be assigned to either iCBT alone or iCBT with the main carer also having access to the same content based on their preference. The primary outcome measure is the Hospital Anxiety and Depression Scale, and alongside secondary measures such as Cancer-Related Quality of Life, Breast Cancer Worry Scale, Brief COPE, and Medical Outcomes Study Social Support Survey will be completed by the survivors at baseline, post-treatment, and 2-month follow-up. Survivors who have carers will also complete Survivor-Carer Cancer Communication and Relationship Quality measures to provide insights into the effects of carer access. To assess the acceptability and satisfaction with the programme, survivors and their main carers will fill out the Helpful Aspects of Therapy Form (HAT) and Satisfaction with Online Treatment (SAT). Programme effectiveness and the effects of carer access on primary and secondary outcome measures will be evaluated on intention-to-treat and per-protocol basis using Linear-Mixed-Models.

**Discussion:**

This is the first trial comparing the effectiveness and acceptability of iCBT with and without carer access for depression and anxiety among breast cancer survivors. The findings of this study will provide novel data on the acceptability of iCBT programmes for breast cancer survivors and the impact of carer access on them and their carers.

## Highlights

• Depression and anxiety are common among breast cancer survivors, and treating them is critically important.
• Carer support is an important determinant of breast cancer survivors’ psychological distress and well-being.
• Effects of carer access to the survivors’ treatment programme will be explored.
• iCBT alone will be compared with iCBT with the main carer access and Treatment-As-Usual.
• A primary outcome of depression and anxiety symptoms will be assessed.

## 1 Background

Improvements in the early detection and treatment of cancer have resulted in an increasing number of people living with cancer for longer periods (Niedzwiedz et al., 2019; Simard and Savard, 2009). Research shows that clinical levels of depression and anxiety are common problems among people as they adjust to the illness (Baqutayan, 2012; Croyle and Rowland, 2003; Fann et al., 2008; Hopko et al., 2016b; Puigpinós-Riera et al., 2018; Reich et al., 2008; Spiegel and Riba, 2015), and symptoms of distress remain for some patients 20 years after the initial treatment (Hegel et al., 2006). Depression and anxiety symptoms in people living with breast cancer are associated with poorer physical health, more pain and fatigue, more substance use, poorer quality of life, less acceptance and compliance with adjuvant treatments, higher prevalence of metastasis, higher risk of relapse and mortality, as well as lower survival time (Chida et al., 2008; Colleoni et al., 2000; Hjerl et al., 2003; Hopko et al., 2008; Hopko et al., 2014; Hopko et al., 2016a; Reich et al., 2008; Reuter et al., 2006; Reyes-Gibby et al., 2012; Somerset et al., 2004; Spiegel and Riba, 2015). Therefore, treating the distress associated with the illness is of critical importance.

Women with breast cancer often deal with this stressful experience by turning to their partners or spouses (Manne et al., 2016; Salakari et al., 2017), or their close relatives, and friends for emotional support (Ockerby et al., 2013); usually one of these people will assume a caregiver role (Tiete et al., 2020). Social support from significant others is one of the most important determinants of patients' positive psychological adjustment and lower psychological distress (Helgeson et al., 2004; Hughes et al., 2014; Ng et al., 2015; Thompson et al., 2017). Open and mutual communication about cancer-related issues and concerns between patients and carers has been associated with less distress and greater relationship satisfaction for both patients and their partners (Manne et al., 2006). A recent randomised controlled study suggested that encouraging cancer patients and carers to express their personal cancer-related concerns helps improve their coping ability with the illness (Tiete et al., 2020). Active participation of the carer in the intervention through a couple-based intervention might help improve the quality of life, psychological distress, and relationship functioning of couples coping with breast cancer as demonstrated by a systematic review (Brandão et al., 2014). However, couple-based interventions for cancer have lower uptake rates than individual-based interventions, especially when both the patient and the carer are required to participate in the intervention simultaneously (Hopkinson et al., 2012; Regan et al., 2013).

Several meta-analyses have established Cognitive Behavioural Therapy (CBT) as clinically effective in the treatment of depression and anxiety among breast cancer patients and survivors (Tatrow and Montgomery, 2006; Xiao et al., 2017; Ye et al., 2018). Its long-term effect on reducing depression and increasing quality of life among breast cancer survivors was also shown in a randomised controlled trial with an 11-year follow-up (Stagl et al., 2015). A CBT intervention teaching adaptive coping skills to manage daily stressors and encourage the use of social resources reduced depression levels among women with breast cancer (Gudenkauf et al., 2015). Consistent with this finding, studies with breast cancer patients revealed that active coping strategies were associated with better adjustment and quality of life, whereas avoidant coping strategies were associated with poorer psychological well-being and decreased quality of life (Holland and Holahan, 2003; Kershaw et al., 2004; Moorey and Greer, 2012).

However, access to evidence-based CBT treatments remains challenging (Etzelmueller et al., 2018) due to the costs, distance to service locations, perceived personal stigma of mental disorders and treatments, lack of trained therapists, inadequate treatment, and delayed treatment provision (Kessler et al., 2001; Mohr et al., 2010; Wang et al., 2007; Wittchen et al., 2011). Besides, many people living with cancer who could benefit from psychological support remain untreated because of the under-recognition of the need for psychosocial care by their primary oncology team (Fallowfield et al., 2001) and even when recognised, the lack of professionals to provide psychological support to cancer patients is hindering (Jacobsen and Jim, 2008).

An internet-delivered cognitive behavioural therapy (iCBT) intervention focusing mainly on alleviating survivors' depression and anxiety symptoms, but also open for carers (e.g., spouses, family members, and friends) to access may overcome these aforementioned challenges. It would allow breast cancer survivors and their carers access to the programme at a time and place convenient to them regardless of their location; furthermore, it may reduce concerns about stigma and privacy (Andersson and Titov, 2014; Donker et al., 2015; Griffiths and Christensen, 2007). Giving carers access to the iCBT content, which does not require simultaneous log-in by survivors and their informal carers might be more appealing for this population, considering carers' difficulty to find time due to other commitments (Regan et al., 2013). Carer access would help carers to better understand what survivors have been going through and widen their knowledge about cancer-related issues from the survivors' perspective. Having the same knowledge through accessing to same content may also encourage them to openly discuss their cancer-related concerns and practice the coping skills that they learned together to deal with psychological distress. This may improve survivors' perceived social support, cancer-related communication, and relationship quality between survivors and carers; this may result in a further decrease in survivors' depression and anxiety symptoms in combination with the treatment effect.

### 1.1 Effectiveness of iCBT in the treatment of depression and anxiety in breast cancer

iCBT is a promising approach that can be used to provide information and support to cancer survivors on managing unhelpful thinking and behaviours, normalising feelings, and thereby relieving distress (Igelström et al., 2020). Research shows that iCBT interventions are highly effective in the treatment of major depression (Andersson and Cuijpers, 2009; Hedman et al., 2012; Wright et al., 2019), subthreshold depression (Buntrock et al., 2015; Spek et al., 2007b), anxiety (Richards and Richardson, 2012; Spek et al., 2007a; Titov et al., 2009), as well as for comorbid depression and anxiety (Newby et al., 2013) in the general population. In a meta-analysis, guided self-help and face-to-face therapy for depression and anxiety disorders had comparable effect sizes at post-treatment and follow-up periods for up to 1 year, suggesting no statistically significant differences (Cuijpers et al., 2010). Guided iCBT interventions (supported by coach or therapist) for depression and anxiety have been found as more cost-effective than waiting-list, treatment-as-usual, attention-control, telephone counselling, group cognitive behaviour therapy, and unguided internet CBT (Donker et al., 2015; McCrone et al., 2004).

Effectiveness studies of iCBT interventions for depression and anxiety among cancer patients and survivors are only recently emerging, and most studies have included people with all cancer types rather than focusing only on one type of cancer (e.g. Beatty et al., 2016; Duffecey et al., 2013; Murphy et al., 2019; Willems et al., 2017). A randomised controlled trial provided support for the short-term effectiveness of an online intervention that teaches skills to manage psychosocial and lifestyle-related issues in reducing depression and fatigue among cancer survivors (Willems et al., 2017). Similarly, a randomised controlled trial of iCBT for clinical depression and/or anxiety revealed that cancer survivors in the iCBT group showed significant reductions in anxiety and depression symptoms, general distress, fear of cancer recurrence, and significantly higher quality of life at post-treatment compared to the survivors receiving treatment-as-usual (Murphy et al., 2019). A recent review of online interventions (including web-based, blended care, telehealth, mHealth, and other online interventions) aimed at reducing psychological distress in cancer patients demonstrated mixed findings (Willems et al., 2020). The findings partially supported the reduction of psychological distress and depression following the intervention; however, the evidence for reducing anxiety was limited. Given the limited number of studies, more studies and larger effectiveness trials are needed.

In qualitative research, iCBT has also been demonstrated as acceptable among cancer patients who used the programme. Early-stage cancer patients and survivors with depression and/or anxiety found the iCBT programme acceptable in terms of the format of internet-delivery and the user-friendly nature of the material; overall, participants had good engagement with the programme (Karageorge et al., 2017). Both recent cancer survivors and providers working in cancer care in Canada reported that therapist-guided iCBT is acceptable for their needs (Alberts et al., 2018). Likewise, cancer patients (breast, colorectal, and prostate) undergoing cancer treatment and concurrently experiencing depression and/or anxiety symptoms found it beneficial to have direct access to the online intervention programme as they have been experiencing time constraints in everyday life (Igelström et al., 2020). However, no study has investigated the attitudes of breast cancer survivors and/or their main carers toward iCBT programmes.

Internet interventions designed specifically for people living with breast cancer have so far focused on insomnia (Dozeman et al., 2017; Zachariae et al., 2018), sexual dysfunction (Hummel et al., 2015), body image (Hummel et al., 2018), treatment-induced menopausal symptoms (Atema et al., 2019; Atema et al., 2016), and fatigue (Abrahams et al., 2017). The number of iCBT studies targeting the symptoms of depression and anxiety in people living with breast cancer is indeed very limited (Carpenter et al., 2014; Owen et al., 2005) and the available interventions were self-guided, i.e., no supporter or therapist provided feedback to the users on the programme. However, the findings of the available studies demonstrated promising results. For example, Carpenter et al. (2014) found that 10-week self-guided iCBT is effective in improving self-efficacy for coping with cancer, regulating negative mood, and lower levels of cancer-related post-traumatic stress symptoms compared to the control group among breast cancer patients. Owen et al. (2005) examined the effects of a self-guided internet coping group among women living with early-stage breast cancer in a randomised pilot study and found that women with low self-reported health status at baseline had greater improvement in their perceived overall health after the treatment. However, to the best of our knowledge, there is no available guided internet intervention aiming to alleviate depression and anxiety symptoms specifically in breast cancer survivors.

Despite the critical influence of the social environment, specifically the perceptions of partner and carer support, on breast cancer patients' psychological adjustment (Gremore et al., 2011; Helgeson and Cohen, 1996; Manne et al., 2014; Manne et al., 2005; Sormanti and Kayser, 2000), only one randomised controlled trial included male partners of young women with breast cancer in an online intervention with a couple-based approach; no findings have been published yet (Fergus et al., 2015). To date, no trial on internet interventions has evaluated whether giving main carers access to the breast cancer survivors' programme has any effect and whether giving carers access decreases survivors' psychological distress further. There are some internet interventions available for caregivers of different populations such as the caregivers of women with breast cancer (Levesque et al., 2018), depressed patients (Blom et al., 2015; Bijker et al., 2017), and people with dementia (Meichsner et al., 2019). However, none of these studies targeted breast cancer survivors' well-being or psychological distress but focused only on improving caregivers' well-being.

### 1.2 Adaptation of the iCBT programme for breast cancer survivors

Space from Depression and Anxiety is an 8-week CBT based online intervention programme, designed originally for the treatment of depression and anxiety symptoms in the general population and adapted for the breast cancer context for this study. The original Space from Depression programme (without the anxiety module) has been found effective for adults with depressive symptoms in the general population, with a large effect size at post-treatment (d = 0.91; Richards et al., 2015). The differences between treatment and waiting-list control group also yielded statistically significant results, with a moderate to large effect size (d = 0.50), and these improvements on the outcomes were maintained at 6-month follow-up. Most participants reported that they were satisfied with the programme, felt supported, reported positive gains and impact, and perceived these as lasting effects (Richards et al., 2016). However, the programme content (e.g., examples and personal stories) was not suitable for the breast cancer survivor and carer context, thus adaptation was required. In a recent randomised waitlist-controlled effectiveness and cost-effectiveness trial of digital interventions, it was found that 8-week iCBT for depression and anxiety is not only effective but also potentially cost-effective in the long-term within the improving access to psychological therapies programme (IAPT) in the UK (Richards et al., 2020).

### 1.3 Space in breast cancer from depression and anxiety

For the adaptation of the programme, *Space in Breast Cancer from Depression and Anxiety*, semi-structured interviews were conducted with 5 women living with breast cancer and 3 main carers (sister, partner, friend). Based on their feedback on the original programme and the findings in the literature, the programme has been adapted for women with breast cancer and their main carers by the principal researcher (SA). After the adaptation of the content, one woman living with breast cancer, who completed the treatment, evaluated all the personal stories, and stories were tailored accordingly. Then, a clinical psychologist, who is an expert in CBT and psycho-oncology reviewed the first three modules of the adapted programme and gave feedback. Next, the primary researcher reviewed the changes and edited the whole programme content based on the received feedback. Afterward, another clinical psychologist who is an expert in CBT reviewed the adapted content including all eight modules and the programme content was updated accordingly.

The adaptation included combining the first two modules, updating and checking the relevance of research-based information in the breast cancer context, adding information on (i) breast cancer (definition, risk factors, its effects on well-being), (ii) the manifestation of depression and anxiety symptoms in breast cancer, (iii) the effectiveness and use of CBT for breast cancer, (iv) adapting physical activities for patients who have finished their treatment, and (v) how to support as a carer. Furthermore, the quiz questions and answers were revised to make them focused on the module content.

The personal stories in the programme were rewritten for breast cancer survivors, which were mostly inspired by the experiences of the interview participants and the clinical knowledge of the primary researcher. This was done to help survivors and carers relate to personal stories. In total 6 personal stories were prepared: 2 representing single women living with breast cancer, 2 for informal carers of the survivors, and 2 for survivor-carer cancer-related relationship problems. Careful attention was paid to ensure that the stories accurately portrayed different experiences. As the fear of cancer returning and progressing in the same organ or another part of the body is one of the most common and distressing concerns among cancer survivors (Niedzwiedz et al., 2019; Simard and Savard, 2009; Yang et al., 2019), the fear of recurrence was also captured in the stories in addition to the examples in other sections of the programme. Photos in the personal stories section of each module were also replaced with images that best suited the new characters created.

Moreover, all the examples were checked and replaced with breast-cancer specific examples in sections such as thinking errors, thoughts-feelings-behaviour (TFB) cycles, and worry cycles. For example, the TFB cycle examples did not address the common negative thoughts experienced by breast cancer patients; thus, they were changed with the ones such as “I can't cope with breast cancer” or “My cancer will come back”. Furthermore, there were other examples in the programme that did not fit the new context. For example, an example of catastrophising in the thinking errors section was “Stumbling over a few words when giving a presentation and then thinking the whole thing was a mess”, which did not match the new context; therefore, these were modified, for example, with “although your doctor says your prognosis is good, you still believe that you can't recover from cancer” to reflect common thinking patterns in the context of breast cancer.

Information on fatigue was added as suggested by the patients in Alberts et al.'s study (2018). Information on emotional expression and effective listening was also added as it was the main difficulty mentioned by most of the participants during the interviews and was not included in the original version of the programme. In addition, distraction techniques for coping with difficult situations were introduced in the Challenging Thoughts module as some of the negative thoughts that patients with advanced breast cancer experience (e.g., the possibility of treatment failure, the possibility of death) may indeed have a realistic basis (Moorey and Greer, 2012). Content that is suitable for breast cancer patients was kept as it is.

### 1.4 Aims of the study

The present pilot study has six aims:

a) To evaluate the effectiveness of an adapted iCBT on the treatment of breast cancer survivors' depression and anxiety symptoms in response to the treatment
b) To determine any changes in breast cancer survivors' quality of life, coping, fear of cancer recurrence in response to the treatment
c) To evaluate any changes in breast cancer survivors' perceived social support, cancer communication, and relationship quality in response to the carer access to survivors' treatment content
d) To evaluate any changes in carers' cancer communication and relationship quality in response to their access to survivors' treatment content
e) To compare the effects of an adapted iCBT with and without main carer access on breast cancer survivors' depression and anxiety symptoms
f) To evaluate the helpful aspects of the iCBT intervention and satisfaction with the programme among breast cancer survivors and their main carers

Therefore, breast cancer survivors will be randomly assigned to either iCBT intervention or treatment-as-usual control. The survivors assigned to the iCBT intervention group and who have a carer will be asked their preference to give their carers access to the intervention programme content. The iCBT group will be allocated to either iCBT alone or iCBT with carer access based on their preference. Therefore, there will be 3 groups in total.

Group 1: iCBT alone

Group 2: iCBT with carer access

Group 3: Treatment-as-usual control

### 1.5 Hypotheses and research questions

Hypothesis about the primary outcomes:

**H1** Survivors in both treatment groups (Group 1 and Group 2) are expected to show a greater reduction in depression and anxiety symptoms than survivors in the TAU (Group 3) at post-treatment and 2-month follow-up.

Hypotheses about the secondary outcomes:

**H2** Survivors in both treatment groups (Group 1 and Group 2) are expected to have greater improvements in cancer-related quality of life, coping, and fear of recurrence compared to survivors in the TAU (Group 3) at post-treatment and 2-month follow-up.

**H3** Survivors in both treatment groups (Group 1 and Group 2) are expected to use more active coping strategies than survivors in the TAU (Group 3) at post-treatment and 2-month follow-up.

**H4** A reduction in depression and anxiety symptoms of survivors in both treatment groups (Group 1 and Group 2) is expected to be mediated by the change in coping strategies after controlling for depression and anxiety levels at baseline.

**H5** An improvement in cancer-related quality of life in both treatment groups (Group 1 and Group 2) will be mediated by the change in coping strategies after controlling for cancer-related quality of life at baseline.

The effects of carer access will be explored using the following research questions:

**RQ1.** Is there a difference in depression and anxiety scores between iCBT alone (Group 1) and iCBT with the carer access (Group 2) at post-treatment after controlling for baseline depression and anxiety scores?

**RQ2.** Is there a difference in perceived social support between iCBT alone (Group 1) and iCBT with the carer access (Group 2) at post-treatment after controlling for baseline perceived social support?

**RQ3.** Is there a difference in survivor-carer relationship outcomes (cancer-related communication quality and relationship quality) of survivors in iCBT with carer access between baseline and post-treatment?

**RQ4.** Is there a difference in survivor-carer relationship outcomes (cancer-related communication quality and survivor-carer relationship quality) of carers between baseline and post-treatment?

## 2 Methods and design

### 2.1 Study design

**Fig. 1:**
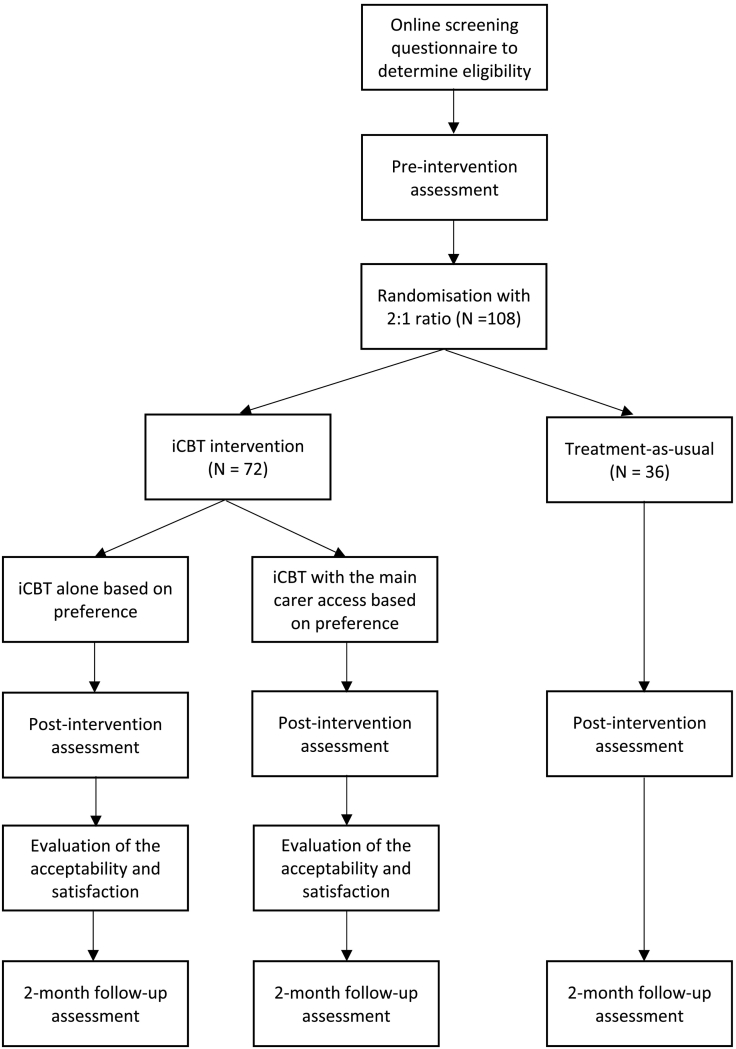
Participant flow chart.

This study is comparing 7-module iCBT with guidance against iCBT with main carer access and treatment-as-usual control among women living with breast cancer who completed their treatment. Both immediate treatment groups will be followed up until 2 months post-treatment. This is a pragmatic pilot trial as survivors' allocation to the iCBT alone or iCBT with the carer access group will be based on survivors' preference. Therefore, the effects of being in the iCBT with the carer access arm are examined in an exploratory manner using research questions rather than confirmatory hypotheses testing. Fig. 1 shows the outline of the trial design in detail.

### 2.2 Study setting and participants

Participants will be recruited online from across Ireland and will be screened for eligibility. Inclusion criteria for survivors are (1) being female, (2) having completed the active breast cancer treatments such as chemotherapy, radiotherapy, and surgery (participants could be on hormone therapy) and being cancer-free, (3) being at least mildly confident with using the internet, and (4) being at least mildly confident in reading and writing in English.

Inclusion criteria for main carers are (1) currently caring or have cared for a woman living with breast cancer (such as a partner, spouse, friend, or relative), (2) being at least mildly confident with using the internet, and (3) being at least mildly confident in reading and writing in English.

Exclusion criteria for breast cancer survivors and carers are (1) current suicidal ideation or intent, (2) current alcohol or drug misuse, (3) enduring mental health disorders such as schizophrenia, psychosis, and bipolar disorder, and (4) currently being in psychological treatment for depression or anxiety. As it is a pilot study, no limit has been set for the time since the treatment completion or cancer stage as an exclusion criterion for survivors.

### 2.3 Recruitment

Participants will be recruited through online cancer support groups, cancer research email listings, flyers, posters, and internet social media advertising (Facebook and Twitter). A Qualtrics link will be provided on the advertisement and via email. All survivors who show an interest in this trial will be able to read the Information Leaflet and sign the informed consent through the provided Qualtrics survey link.

### 2.4 Sample size

The G-Power software programme was used to carry out power analysis and determine the sample size needed for three-group, pre-test, post-test, and follow-up design. The data from a randomised controlled trial of an iCBT programme for depression and anxiety among cancer survivors revealed a large within-between interaction effect size for the HADS-Total after comparing the pre-treatment and post-treatment scores of the iCBT and the TAU (Murphy et al., 2019). To calculate the sample size, we powered for the changes in the primary outcome (depression and anxiety symptoms, measured by the HADS-Total) when comparing the treatment groups (iCBT alone and iCBT with the carer access) with the TAU. Assuming a medium effect size (*f* = 0.30) for the pre, post, follow-up comparisons between iCBT alone, iCBT with carer access, and TAU with a power of 0.80 and significance level of α = 0.05, the sample size is calculated to be 27 breast cancer survivors in each group and 81 in total (54 in the iCBT group and 27 in the TAU group using 2:1 ratio). Considering the 25% attrition (Richards et al., 2018), 108 breast cancer survivors in total (*n* = 72 for iCBT and = 36 for TAU) will be recruited.

### 2.5 Randomisation

Eligible participants will be randomly assigned to either iCBT intervention (n = 72) or treatment-as-usual control (*n* = 36) with a 2:1 ratio. Those who are assigned to iCBT group and have carers will be asked whether they will participate with or without their main carer. Participants will be assigned to iCBT alone or iCBT with carer access based on their preference and informed immediately about their group assignment. Survivors in the iCBT alone group (Group 1) will be using the iCBT programme by themselves, and survivors in the iCBT with the main carer access group (Group 2) will be using the same programme, but their main carers will also have access to the programme content using a separate username and password. Participants in the treatment-as-usual control group (Group 3) will continue their usual care and will not receive the iCBT programme during this study. They will fill out the same measures as the other two groups at pre-treatment, post-treatment, and 2-month follow-up.

### 2.6 Procedure

The link on the advertisement will direct potential participants to a Qualtrics page to complete the informed consent, screening questionnaire assessing inclusion criteria, demographic information, and health status. All participants will be responsible for self-screening as their eligibility will be determined based on their responses to the questions set-up in Qualtrics. The survey is designed to automatically exclude respondents who select “yes” to the questions asking current suicidal ideation or intent, current alcohol or drug misuse, and organic mental health condition (such as schizophrenia, psychosis, and bipolar disorder), and current psychotherapy for depression or anxiety. Potential participants who responded yes to the questions regarding current suicidal ideation and intent will see a message providing contact details for recommended support services on the screen. Eligible survivors will be able to continue with baseline questionnaires (Time 1) assessing depression and anxiety symptoms, quality of life, fear of recurrence, coping, perceived social support, relationship quality, and cancer-related communication. After filling out the questionnaires, eligible participants will be automatically randomised and those who prefer to participate with their carer (iCBT with the main carer access) will be asked to provide their carers' email address on the Qualtrics page. Carers will be contacted through the email address provided by breast cancer survivors and asked to complete the informed consent, screening questionnaire, and survivor-carer cancer communication, and relationship quality measures through a separate Qualtrics link. All survivors and carers will be reassessed online at the end of the intervention (7 weeks after they started, Time 2), and at 2-month follow-up (Time 3). At the end of the intervention, survivors and their main carers in both treatment groups will be asked about their perspectives on the helpful aspects of the programme and their satisfaction with the programme.

### 2.7 iCBT intervention: space in breast cancer from depression and anxiety

*Space in Breast Cancer from Depression and Anxiety* is a seven module CBT-based intervention for depression and anxiety adapted specifically for women breast cancer survivors and their informal main carers. The programme was designed to alleviate depression and anxiety symptoms through the use of CBT skills such as activity planning (behavioural activation), understanding the role of feelings, identification of unhelpful or irrational thinking patterns, generating more realistic and helpful thoughts by changing their perspective (cognitive restructuring), setting up a worry time, and differentiating practical and hypothetical worries.

It consists of 7 modules completed ideally over 7 weeks and each takes approximately an hour to complete. A brief description of the programme content in each module can be seen in Table 1. There are also additional resources and tools available in the programme such as a journal, mindfulness and relaxation audio exercises, activity scheduling, activities list, and mood monitor. Each module in the programme includes quizzes, personal stories, exercises, and a summary.

**Table 1:** Content of the intervention programme.

Module	Description
1. Getting started	This module provides information about breast cancer, depression, and anxiety, and why do they occur in breast cancer survivors. It also introduces the basics of CBT.
2. Understanding feelings	This module introduces emotions, outlines the function of them, and how they are related to our physical body reactions. It also encourages users to express their emotions and includes examples about how to do it tactfully.
3. Boosting behaviour	Boosting behaviour is a module focusing on behavioural activation as a way to improve users' mood. It helps them to identify and plan pleasurable activities that will give them a sense of achievement and help them feel better.
4. Spotting thoughts	This module aims to help users identify their unhelpful, negative thinking patterns, distorted thinking errors, and builds their own TFB cycles.
5. Challenging thoughts	Challenging Thoughts module helps users to identify and challenge their unhelpful hot thoughts and find an alternative thought that is more balanced and realistic.
6. Managing worry	This module explains the role of worry in anxiety and introduces the worry cycle. It also introduces techniques to manage both real and hypothetical worries.
7. Bringing it all together	This module encourages users to reflect on what information and skills they have learned and helps them to make a plan to stay well by watching out for personal warning signs and maintaining social support.

### 2.8 Supporters

As this is a guided iCBT intervention, each survivor will be assigned a supporter, who provides weekly feedback on their progress on the programme. The supporters are masters' level students in applied psychology who have completed an undergraduate degree in Psychology. They were given an online health supporter training by an experienced psychologist from SilverCloud Health. The training took 1.5 h and the aim was to make supporters to become familiar with the evidence base of iCBT, to provide them a working knowledge of the platform being used, and help them communicate effectively with the users through demonstrating how to write reviews. The supporters were also given psycho-education training on breast cancer and its psychological impacts by the principal researcher (SA) before starting their role as a supporter. Supporters will be provided weekly supervision by the researchers (SA and DH).

Each supporter will provide asynchronous weekly feedback on the platform to their assigned users. Feedback will take 10 to 15 min per participant for each session, which will consist of motivating users to keep using the programme, providing feedback on their progress, and suggesting the use of specific helpful tools in the programme. Supporters will schedule a specific time for feedback each week for a period of seven weeks so that survivors will know when they will get the feedback.

### 2.9 Measures

#### 2.9.1 Screening measure

##### 2.9.1.1 Sociodemographic and clinical history questionnaire

The Sociodemographic and Clinical History Questionnaire is an instrument developed to be used as a screening measure for an online CBT intervention (Richards et al., 2013). It includes questions such as alcohol or drug misuse, suicidal thoughts, confidence in using the internet. It also measures whether they have a diagnosis of an organic mental health condition (i.e., schizophrenia, psychosis, and bipolar disorder) and whether they previously received psychotherapy for depression or anxiety. The questionnaire is adapted for the present study with additional health-related questions such as diagnosis of breast cancer, length of time since the diagnosis, received medical treatments (chemotherapy, radiotherapy, or hormonal therapy), and main carer related questions such as whether they have someone who supports them.

#### 2.9.2 Primary outcome measure

##### 2.9.2.1 Hospital anxiety and depression scale (HADS)

HADS is a 14-item self-report questionnaire, which is designed to measure anxiety and depression symptoms in medical settings (Zigmond and Snaith, 1983) will be used as a primary outcome measure. It consists of two 7 item subscales: depression (HADS-D) and anxiety (HADS-A). Each item is rated on a 4-point scale ranging from 0 “Not at all” to 3 “Most of the time”. After reverse scoring eight of the items, HADS-D and HADS-A subscale totals can be summed, giving a maximum score of 21 for each subscale. The HADS-T is the total score of depression and anxiety subscales, which will be used in the present study. High scores represent higher psychological distress. The HADS-T was chosen over other depression and anxiety measures as it measures anxiety and depression without relying on somatic symptoms such as fatigue or insomnia, which are experienced by cancer survivors as a result of their illness and/or medical treatment. The HADS is validated and extensively used in the measurement of distress among cancer survivors (Vodermaier et al., 2009; Mitchell et al., 2010).

#### 2.9.3 Secondary outcome measures

##### 2.9.3.1 Cancer-related quality of life (EORTC QLQ-C30)

To assess the overall health-related life quality of cancer survivors, a single item from the European Organization for Research and Treatment of Cancer Quality of Life Core Questionnaire (EORTC) will be used (Aaronson et al., 1993). It is a 30-item scale that assesses various facets of health-related quality of life; only the final item “How would you rate your overall quality of life during the past week?” will be used in the current study. Responses to this single item are rated on a 7-point scale ranging from 1 “very poor” to 7 “excellent”.

##### 2.9.3.2 Breast Cancer Worry Scale (CWS)

The six-item version of the Cancer Worry Scale (CWS) (Custers et al., 2018) will be used to assess breast cancer survivors' concerns about cancer recurrence and the impact of these concerns on their daily functioning. The scale was originally to measure fear of developing cancer in women at risk of hereditary cancer (Lerman et al., 1991). The items include “How often have you thought about your chances of getting cancer again?”, “Have these thoughts affected your mood?”. All items are rated on a 4-point scale ranging from 1 “never” to 4 “almost always”, with scores ranging from 6 to 24 and higher scores indicating more frequent worries about breast cancer. The 6-item version of the CWS has good construct, convergent and divergent validity, and high internal consistency to detect fear of cancer recurrence in breast cancer survivors, with a Cronbach alpha coefficient of 0.90 (Custers et al., 2018).

##### 2.9.3.3 Brief coping orientation to problems encountered (brief COPE) inventory

The Brief COPE is a 28-item measure, which assesses strategies used for coping with a stressful life event (Carver, 1997). This inventory is an abbreviated version of the full 60-item COPE Inventory (Carver et al., 1989) and assesses 14 different coping strategies: self-distraction, active coping, denial, substance use, emotional support, instrumental support, behavioural disengagement, venting, positive reframing, planning, humour, acceptance, religion, and self-blame (Carver, 1997). It has response options range from (1) I haven't been doing this at all to (4) I have been doing this a lot. It measures both adaptive and maladaptive coping strategies and is commonly used for measuring coping with health-related conditions (Rand et al., 2019). In the present study, the instructions were modified to ask how participants had been coping with breast cancer-related stress in their life. Item samples are: “I've been turning to work or other activities to take my mind off things”, “I've been concentrating my efforts on doing something about the situation I'm in”. This scale will be used to measure potential changes in survivors' ways of coping with stress related to breast cancer before and after the intervention. The Brief COPE has been demonstrated to be reasonably reliable with Cronbach alpha levels ranging from 0.50 to 0.90 for each subscale (Carver, 1997; Rand et al., 2019). In the present study, instead of 14 subscales, 2 coping dimensions that assess efforts to move toward goals (active coping) and to disengage from goal pursuits (avoidant coping) will be used (Kershaw et al., 2004; Rand et al., 2019).

##### 2.9.3.4 Medical outcomes study social support survey (MOS-SSS)

Survivors' perceived support will be measured using the Medical Outcomes Study social support survey (MOS; Sherbourne and Stewart, 1991). The scale was originally developed to measure the perceived availability of functional social support among people with chronic illness. The original scale assesses four dimensions of social support: (1) emotional/informational support (the expression of positive affect, empathetic understanding, and the encouragement of expressions of feelings, and the offering of advice, information, guidance, or feedback), (2) tangible support (the provision of material aid or behavioural assistance), (3) positive social interaction (the availability of other persons to do fun things with you), and (4) affectionate support (involving expressions of love and affection). In the present study, only the emotional/informational support subscale (8 items) will be used as carer access may only influence the emotional/informational social support perceptions of survivors. Survivors will be asked how true each statement was based on the support available to them during the past 7 days. Sample items include: “if you need it, how often is someone available you can count on to listen to you when you need to talk?”, “if you need it, how often is someone available to give you good advice about a crisis?”. The responses to all items range from “none of the time” to “all of the time”; higher scores indicating better perceived social support. It had excellent reliability with 0.97 for the overall scale and subscales with a Cronbach's alpha of 0.96, 0.94, 0.91, and 0.92, respectively. This scale has been used in many studies with breast cancer patients (Chang et al., 2019; Ganz et al., 2003; Kornblith et al., 2003; Kroenke et al., 2013; Sousa Rodrigues Guedes et al., 2020).

##### 2.9.3.5 Survivor-carer cancer communication

Cancer communication difficulties between survivors and carers will be measured by 6-items drawn (Family communication subscale) from the 30-item measure of Patient-Family Discord (Francis et al., 2010; Siminoff et al., 2006). This measure was chosen as the items are specific to cancer communication and expression of feelings about cancer, which were addressed during the adaptation of the iCBT intervention. Some examples of the items are: “My family does not really listen when I talk about my cancer”, “I avoid talking about cancer to my family because I don't want to upset them”. The word family was changed with the carer in the breast cancer survivors' version as this study is focusing only on the carer and survivor relationship. In the carer version of the scale, items were changed as “She does not really listen when I talk about her cancer”, “I avoid talking about cancer to her because I don't want to upset her”. The items are rated on a 5-point scale response ranging from 1 to 5 (1 = strongly agree, 5 = strongly disagree) in the present study. The scale demonstrated a reliability coefficient of 0.63 (Francis et al., 2010).

##### 2.9.3.6 Survivor-carer relationship quality

Survivors and carers will be asked to rate the quality of their relationship with each other on a scale ranging between 0 and 10. A score of 10 represents the excellent quality of relationship and 0 represents the very poor quality of the relationship. This single item scale measuring the relationship quality between survivors and carers will allow them to base their judgments on aspects of their relationship that are most important for them (based on Cantril, 1965; Kuijer et al., 2004; Rottmann et al., 2015).

#### 2.9.4 Other measures

##### 2.9.4.1 Helpful aspects of therapy form (HAT)

The HAT assesses the most helpful and hindering effects for survivors during the therapy (Burke et al., 2019; Llewelyn, 1988). It asks survivors to describe, in their own words, what was most helpful and what was least helpful in each module for them. It can be anything that participants, the module, or supporters said or did. To clarify their associated impacts, the scale also asks them to describe what made the event helpful or hindering for them.

##### 2.9.4.2 Satisfaction with online treatment (SAT)

The SAT assesses satisfaction with online treatment (Richards and Timulak, 2013). The first part consists of questions about the use of the computer to access treatment, how easy it was for them to use online treatment, whether the treatment they received would have a lasting effect on them, and whether they would specifically recommend online treatment to others. Responses are reported on a scale ranging from “Agree very strongly” to “Disagree very strongly”. The second part consists of two qualitative questions asking participants to describe what they most liked and least liked about the treatment.

### 2.10 Planned analyses

#### 2.10.1 Baseline comparisons

All data will be organised and prepared in the Statistical Package for the Social Sciences (SPSS). Demographic information and characteristics of the participants (i.e., age, education level, breast cancer stage, presence of the main carer) will be analysed using descriptive statistics. Group differences in demographic and clinical characteristics at baseline will be analysed with the Chi-squared test for categorical variables, and analysis of variance (ANOVA) or *t*-tests for continuous variables.

#### 2.10.2 Effectiveness analyses for primary and secondary outcomes

Linear Mixed Modelling (LMM) will be used to compare pre-treatment, post-treatment, and 2-month follow-up scores of the survivors in three groups (iCBT alone, iCBT with the carer, and TAU control) on the main outcome measure of depression and anxiety as well as secondary outcome measures including cancer-related quality of life, coping, and fear of cancer recurrence. The intention-to-treat (ITT) and per-protocol (PP) approach will be used to test the effectiveness of the treatment groups over the TAU control group. According to the ITT approach, participants who leave the study early or do not comply with the protocol are considered as belonging to the treatment arm they were originally randomised to. On the other hand, PP analytical approach does not include patients who violate the protocol, switch the allocation arm throughout the study, do not adhere to the assigned treatment or do not undergo to the scheduled assessments over time. Thus, it helps capture the “true” exposure to treatment. As suggested, the ITT will be used as the main method of analysis to determine effectiveness as it provides an unbiased estimate to treatment effect, and the PP will be used as a secondary and supportive analysis (Tripepi et al., 2020) to provide complementary information about the iCBT intervention. Restricted maximum likelihood (REML) estimation will be used to account for missing data due to participant drop-out. Significant effects will be followed up using pairwise contrasts comparing mean pre-treatment scores to post-treatment scores. Between-group (Hedges' *g*) and within-group (Cohen's *d*) effect sizes will be calculated using the pooled standard deviation and adjusted for sample size. For Hedges' *g* and Cohen's *d* an effect size 0.20 is considered small, 0.50 moderate, and 0.80 large.

Mediation analyses using PROCESS for SPSS (Hayes, 2013; Hayes and Rockwood, 2017) will be used to test the role of change in coping strategies following the iCBT treatment on survivors' outcomes such as depression and anxiety scores and cancer-related quality of life. The estimates of indirect effects will be generated using the bootstrapping method (*n* = 5000 bootstrap re-samples) and a 95% confidence interval (Preacher and Hayes, 2008). If the 95% confidence interval does not include the value of zero, this supports a claim of mediation, i.e., the effect of the independent variable on the outcome variable is contingent upon the effect of the proposed mediator. The bootstrapping method was preferred over the Sobel test as it does not impose the assumption of normality of the sampling distribution and the sampling distribution of the indirect effect is frequently non-normal.

#### 2.10.3 Analyses exploring the effects of carer access

LMM will be used to explore whether there are differences in depression and anxiety scores and perceived social support of survivors who used the iCBT programme alone and survivors who used the iCBT programme with carers having access at post-treatment. To account for the potential confounds, baseline levels of perceived social support will be controlled when comparing the iCBT alone and iCBT with the carer access groups. Those who have someone to care for them may perceive higher social support prior to the iCBT intervention and the initial levels may influence responses to the iCBT programme. LMM will also be used to evaluate whether there are differences in survivors' and their carers' communication quality and relationship quality scores at post-treatment after controlling for baseline scores.

#### 2.10.4 Qualitative analyses

Responses of survivors and carers to helpful and unhelpful aspects of therapy in the HAT will be qualitatively analysed using thematic analysis with an inductive approach by the primary researcher (SA). Inductive analysis is a process of data coding without relying on a pre-existing coding frame or the researcher's theoretical commitments or analytic preconceptions (Braun and Clarke, 2006). To provide insight into participants' experiences, common themes, and patterns concerning helpful and unhelpful aspects of the programme, and their impact on survivors will be identified in this analysis. After the interpretation of the raw data, codes will be separated into helpful aspects, unhelpful aspects, and their impacts, and then initial themes will be generated, coded, and finally given names with clear definitions. These codes will be reviewed by another researcher who has experience in qualitative analysis to discuss the identified patterns and themes in the dataset. Quantitative data from the SAT will be analysed using descriptive analysis and the qualitative responses on the second part of the scale will be analysed using thematic analyses following the same procedure in the HAT analysis.

## 3 Discussion

The current study evaluates the acceptability and effectiveness of the iCBT intervention, *Space in Breast Cancer from Depression and Anxiety*, adapted specifically to alleviate depression and anxiety symptoms of women who completed breast cancer treatment in Ireland. There are some important gaps in the literature this study aims to fill. First of all, no study has targeted depressive and anxiety symptoms in iCBT interventions specifically with breast cancer survivors with all cancer stages. Although Murphy et al.'s study (2019) evaluated the effects of iCBT on clinical depression and/or anxiety among cancer survivors, they only included survivors with early-stage cancer and their findings may not be generalisable for those living with advanced cancer. Therefore, the current trial is the first randomised controlled trial of an online intervention for depression and/or anxiety in breast cancer survivors with all cancer stages. If proven effective and acceptable for breast cancer survivors and their main carers, this programme might be a good evidence-based alternative for women who do not have access to existing face-to-face evidence-based psychological treatments, given the lack of access to evidence-based psychological treatments (Etzelmueller et al., 2018). For example, survivors who are unable to travel to a therapist's office due to lack of energy or physical limitations (Everts et al., 2015) or who live in rural and remote communities (Griffiths and Christensen, 2007) can benefit from iCBT interventions (Lopez-del-Hoyo et al., 2013).

Secondly, no study to date has examined the effectiveness of including informal carers in the survivors' intervention programme targeting their depression and anxiety symptoms. This pilot trial is novel as it further aims to explore whether giving main carers (i.e., partners, family members, friends) access to survivors' iCBT treatment makes a difference on survivors' anxiety and depression symptoms, social support perceptions, and cancer-related communication quality, and relationship quality with their carers as well as carers' cancer-related communication quality, and relationship quality with survivors. This study will provide novel insight regarding the feasibility of carer access based on the number of survivors who prefers to give their carers access to the iCBT programme. The trial is sufficiently powered for testing the primary and secondary hypotheses, and analyses regarding the effects of the iCBT with the carer access are more exploratory. As the iCBT alone and iCBT with the carer access group assignment is based on survivors' preference due to its exploratory nature, the study may be underpowered for detecting those effects in the exploratory analyses. Therefore, the results of the exploratory analyses will be interpreted with caution.

Another notable strength of this study is that it uses a mixed-methods approach by combining quantitative methods to measure the programme effectiveness with qualitative measures through HAT and SAT to assess the user experiences and satisfaction, which will provide rich information and help us to better understand the nature of the existing relationship between outcomes. If the programme is found feasible and a clinically significant difference is found in depression and anxiety symptoms of survivors, the iCBT programme may be used as an alternative within stepped care of breast cancer patients and survivors, where guided low-intensity interventions are an attractive option (Richards et al., 2018).

## Trial status

The trial recruitment started on 30 October 2020.

## Ethics

Approved 15/07/2020, Trinity College Dublin School of Psychology Research Ethics Committee (School of Psychology, Aras an Phiarsaigh, Trinity College Dublin, Dublin 2, Ireland; +353 (0)1 896 1886; psych.ethics@tcd.ie.

## Declaration of competing interest

Ms. Selin Akkol-Solakoglu and Dr. David Hevey declare no conflicts of interest. Dr. Derek Richards is an employee of SilverCloud Health, developers of computerised psychological interventions for depression, anxiety, stress, and comorbid long-term conditions.
